# Laser-Induced Microstructuring of Polymers in Gaseous, Liquid and Supercritical Media

**DOI:** 10.3390/polym13203525

**Published:** 2021-10-13

**Authors:** Alexey Rybaltovskii, Nikita Minaev, Svetlana Tsypina, Svetlana Minaeva, Vladimir Yusupov

**Affiliations:** 1 D.V. Skobeltsyn Institute of Nuclear Physics, M.V. Lomonosov Moscow State University, Vorob’evy gory, 119991 Moscow, Russia; alex19422008@rambler.ru; 2 Institute of Photon Technologies, Federal Scientific Research Centre ‘Crystallography and Photonics’, Russian Academy of Sciences, ul. Pionerskaya 2, Troitsk, 108840 Moscow, Russia; tsypina@yandex.ru (S.T.); minaeva.svetlana@gmail.com (S.M.); iouss@yandex.ru (V.Y.)

**Keywords:** laser microstructuring, laser formation, polybenzimidazole, bubble structures, polymer film

## Abstract

New results are presented for laser formation—in particular, the “drawing” of microstructures in polymer films using continuous-wave (CW) laser radiation λ = 405 nm with an intensity of 0.8–3.7 kW/cm^2^. The laser drawing was carried out in the polymer system poly-2,2′-p-oxydiphenylene-5,5′-*bis*-benzimidazole (OPBI), which consists of two phases: a solid polymer matrix with formic acid (HCOOH) dissolved in it. The formation of microstructures, including the stage of foaming, was carried out in three media: air, water and a supercritical carbon dioxide medium containing dissolved molecules of the silver precursor Ag(hfac)COD. The morphological features of foam-like track structures formed in the near-surface layer of the polymer films by laser “drawing” are considered. A model of processes is presented that explains the appearance of periodic structures. The key point of this model is that it considers the participation of the photoinduced mechanism of explosive boiling of formic acid molecules dissolved in the polymer matrix. Using Raman spectroscopy, spectra were obtained and interpreted, which relate to different stages in the formation of microstructures in OPBI films. The effects associated with the peculiarities of luminescent microstructures on the surfaces of glasses in close contact with polymer films during laser “painting” in the air have been studied.

## 1. Introduction

Over the past three decades, the formation of various microstructures in polymeric materials has been one of the most demanding problems, since its solution largely dictates the progressive development of such areas as biomedical and chemical technologies, polymer microphotonics and nanoplasmonics [1,2,3,4,5,6,7,8]. In most of the reviews and regular articles presented above, various laser sources are used as a working tool, depending on the goals and materials used. For example, femtosecond laser radiation was used on the surfaces of polymers containing dye impurities [5] to obtain linear luminescent structures, which can be used to address problems associated with polymer microphotonics. In addition, CW CO_2_ lasers are widely used to produce microstructured systems in polymer films [6] containing graphene, which is in demand in modern chemical technologies and microelectronics.

In our works [9,10], the possibility of the formation of luminescent surface microstructures in polymeric materials based on polybenzimidazoles, namely in films of poly-2,2′-p-oxydiphenylene-5,5′-bis-benzimidazole (OPBI), was considered for the first time. They had a unique combination of properties: high thermal and chemical resistance, as well as good mechanical strength [11]. It was shown that the thermal destruction of OPBI films occurs at temperatures above 500 °C [12], making it possible to use this material to create a wide range of products for mechanical engineering.

It should be noted that OPBI can, with some assumptions, be considered a two-phase system since its matrix contains a large amount of physically dissolved formic acid molecules, HCOOH (up to 10–20 wt% [12]). The combination of the latter factor with the presence of a strong increase in the absorption coefficient α in the UV region (in the region of 400–410 nm, the value α ≥ 10^3^ cm^–1^ [9]) makes it possible to use this material for the laser “drawing” of foam-like microstructures on the surface, using available diode continuous laser sources with a wavelength λ = 405 nm. The possibility of the formation of foam-like structures in several polymeric materials using pulsed laser radiation was also reported in [13,14,15].

A distinctive feature of OPBI is that luminescent microstructures from bubble formations can be formed both in the absence of coloring impurities [9,12] and with their particular incorporation into a polymer material [16]. In our works, we reported, for the first time, the presence of an intense luminescent glow arising on the surfaces of drying OPBI films [10], as well as in the regions of the films previously irradiated with focused continuous laser radiation with λ = 405 nm [9]. From [17,18], it is known that the initial solutions of polybenzimidazole molecules in formic acid show pronounced luminescence in the green region of the spectrum, the intensity of which decreases as the concentration of molecules in the solution increases, i.e., as it dries. Note that the effect of the laser formation of foam-like luminescent structures in polymer films based on these molecules has been studied in detail [9]. The appearance of luminescence in such structures on the surfaces of polymer films is explained by a decrease in the effect of concentration quenching [19] in chains of macromolecules containing luminescence centers due to their “stretching” during the formation of bubbles.

To purposefully change the functional properties of such foam-like structures, one can use the technique of supercritical fluid (SCF) impregnation [20] of the volume of the starting material with organometallic compounds, which is widely used for the formation of composite materials; this was also successfully used by our group [8,16] to introduce precursors of silver or trivalent europium into the volume of polymeric and porous materials.

The presented work aims to investigate rarely studied features associated with the influence of the environment on the process of the laser formation of near-surface structures in polymeric materials by the example of OPBI. Publications on this problem have appeared relatively recently [21,22], the authors of which draw attention to the features of laser modification of the polymer surface in media with very different heat capacities—for example, in water and air.

This work aims to study the features of the laser-induced formation of extended structures on the surfaces and in the near-surface layers of OPBI films in gaseous, liquid and SCF media when exposed to CW laser radiation with λ = 405 nm. The results of experiments with samples of polymer films carried out in an air atmosphere, in distilled water and in an environment of supercritical carbon dioxide (scCO_2_) with dissolved molecules of a silver precursor are considered.

## 2. Materials and Methods

To study the processes of the laser-induced formation of surface microstructures, we used OPBI film samples that were 60–80 µm thick [12]. Film samples were synthesized by pouring from a formic acid solution, followed by drying in an air atmosphere for two weeks at a temperature of 23 °C. The residual content of acid molecules in the dried film did not exceed 10 wt%. The linear dimensions of the film samples used in the experiments were approximately 10 × 10 mm.

**Figure 1:**
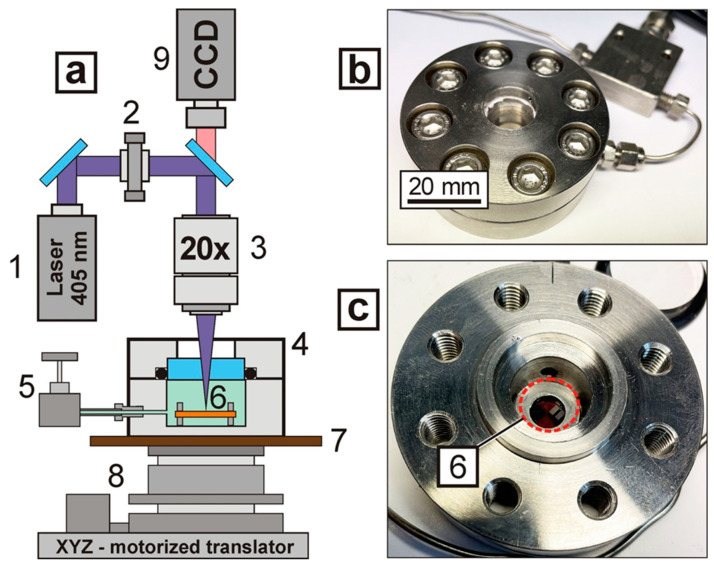
(**a**) Schematic of a setup for laser-induced “drawing” of structures in film samples, in different media: 1—laser source with λ = 405 nm, 2—attenuator, 3—long-focus microlens, 4—high-pressure cell, 5—valve for pressurization/release, 6—film sample, 7—high-pressure cell heater, 8—XYZ motorized translator, 9 – CCD camera; (**b**) A general photograph of a high-pressure optical cell; (**c**) A photograph of the bottom part of the cell, where the red dotted line indicates the position of the OPBI sample clamped between the washers.

For the laser-induced formation of microstructures on the surfaces of polymer films, a laser exposure system (Figure 1a) was used, similar to that presented in [9], with a high-pressure optical cell added. A diode-pumped solid-state laser module with a wavelength λ = 405 nm (MDL-III-405, China) was used as a source of laser radiation. The power P of the laser radiation was set in the range of 13 to 26 mW, which made it possible to change the power density of the focused radiation on the sample surface from 0.8 to 3.7 kW/cm^2^ during the experiments. A long-focus 20X microscopic objective PAL-20-L (OptoSigma, Tokyo, Japan) with n.a. 0.29 and a focal length of 10 mm was used to focus the laser radiation. With the help of a micro-objective, we formed a laser spot with a diameter of 15–30 μm (at the 1/e^2^ level) on the samples. The diameter of the radiation spot on the sample was set by changing the distance from the sample to the objective.

Laser radiation was introduced into the inner volume of the high-pressure cell through a 5 mm thick sapphire optical window. In the inner volume of the cell (~3 cm^3^), the necessary medium was formed: air, distilled water at atmospheric pressure or scCO_2_ medium (temperature 50 °C, pressure 200 bar). The high-pressure cell (Figure 1b) was located on a motorized three-coordinate translator, which allowed it to be moved in space relative to the focusing point of the laser radiation according to a given program.

We used motorized slides OSMS20–85 and OSMS80-20ZF (OptoSigma, Tokyo, Japan) and a Bigtreetech SKR PRO v1.2 controller (Bigtreetech, Guangdong, China), ensuring a sample positioning accuracy of 1 μm. The movement algorithm was set with G-code and the open-source software Pronterface, which created conditions for the laser “drawing” of structures when the sample moved relative to the focusing point of the laser beam. The polymer film sample was placed in the inner volume of the high-pressure cell and fixed between two stainless steel washers that were 2 mm thick (Figure 1c).

For visual control of the process, a digital camera (ToupTek XFCAM1080PHB, Hangzhou, China) with an autofocus module and a telescopic lens was installed in the system, making it possible to obtain an image of the surface of the irradiated sample during the experiments.

When carrying out experiments in the scCO_2_ medium with dissolved precursor molecules, 2 mg of a fine, crystalline, powdery compound, (1,5-Cyclooctadiene)(hexafluoroacetylacetonato)silver(I), [Ag(hfac)(COD)] (CAS Number: 38892-25-0, Aldrich, St. Louis, MO, USA), used in our work with scCO_2_ [23], was placed in the high-pressure chamber together with the sample.

Before starting the “drawing” process, supercritical conditions of CO_2_ were created in the high-pressure chamber for the saturation of the film with the precursor in the SCF solution: pressure 200 bar; temperature 40–50 °C. The exposure time until the beginning of the “drawing” process was 40 min. In the case of irradiation of samples impregnated with a silver precursor in air, free regions of the samples irradiated in the volume of a high-pressure cell were used.

Using the described system for “drawing” linear structures makes it possible to trace the dynamics of their formation during irradiation under various modes. In the experiments, structures were created in the form of a series of separate lines—tracks at different intensities of laser radiation on the film’s surface. For this, the sample was sequentially shifted from line to line, relative to the focal plane of the objective by 50 μm along the vertical axis. As in the case of [9], the distance between the individual lines was 100 µm. The speed of movement of the laser beam over the surface of the sample was 10 mm/s.

Furthermore, experiments were carried out to study the formation of laser-induced microstructures on the back surface of a transparent material when this surface comes into contact with the irradiated polymer film. For this, polymer samples containing a silver precursor were irradiated with laser radiation through a pressed, flat, quartz plate according to the usual scheme. It was assumed that the use of such a scheme would lead to the appearance of microstructures on the back surface of the quartz plate in the form of a kind of “print”. Previously, similar structures were formed in polyimide films [24,25].

To study the development of the formed structures, optical 3D microscopy, scanning electron microscopy (SEM) and Raman spectroscopy were used. With the help of optical 3D microscopy, general information was obtained on the morphology and topography of the formed structures under different modes of formation. Images were recorded using an HRM-300 Series 3D microscope (Huvitz, Gunpo, Korea) with 5X and 10X objectives with a U3CMOS05100KPA digital camera (Touptek, Singapore).

The morphological features of the created microstructures were studied using a Phenom PRO-X scanning electron microscope (ThermoFisher, Amsterdam, The Netherlands). Before being subjected to scanning electron microscopy, the analyzed samples were placed on a standard holder using carbon tape. The silver content in the OPBI matrix was determined using an energy-dispersive microanalysis (EDX) system built into an electron microscope at an accelerating voltage of 15 kV. For the analysis of the results, the software “Element Identification (EID)” was used, which made it possible to determine the sample’s elemental composition both at a specific point and along a plane bounded by a contour.

To obtain cross-sections of samples of the OPBI films with microstructures, they were placed in a two-component epoxy adhesive UHU Plus Schnellfest (UHU, Bühl, Germany). After solidification, a cone-shaped blank was formed from them, which was cut into slices ~5 μm thick using a Leica EM UC7 ultramicrotome (Leica Microsystems, Wetzlar, Germany) and glass knives. The resulting slices and samples of films with a cut surface were also examined using an electron microscope.

Other information on the morphological features of the material and its characteristics at different stages of the formation of microstructures was collected using Raman spectrometry. Raman spectra of film samples were obtained using a Nicolet Almega XR Raman spectrometer (Thermo Scientific, Waltham, MA, USA) using a 50X microscopic objective. A laser source with λ = 532 nm was used to excite Raman scattering. The chosen optical configuration made it possible to record spectra from a spot with a characteristic size of the order of 1–2 µm, with the possibility of precisely targeting the investigated area using a built-in digital camera. These experiments were carried out at a relatively low laser power (<10 mW), which did not introduce irreversible changes in the surface structure of the OPBI samples. To measure the spatial distribution of inhomogeneities in the formed microstructures, a one-dimensional Fourier transform of the brightness distribution of the pixels of the optical image of the analyzed structure along the movement of the optical axis during laser drawing was used [26]. Based on the data obtained, spatial spectra were plotted in the form of curves that illustrated a periodic structure in the analyzed images along the axis of movement of the laser spot.

## 3. Results

### 3.1. Formation of Microstructures in OPBI Films in Air or Water

**Figure 2:**
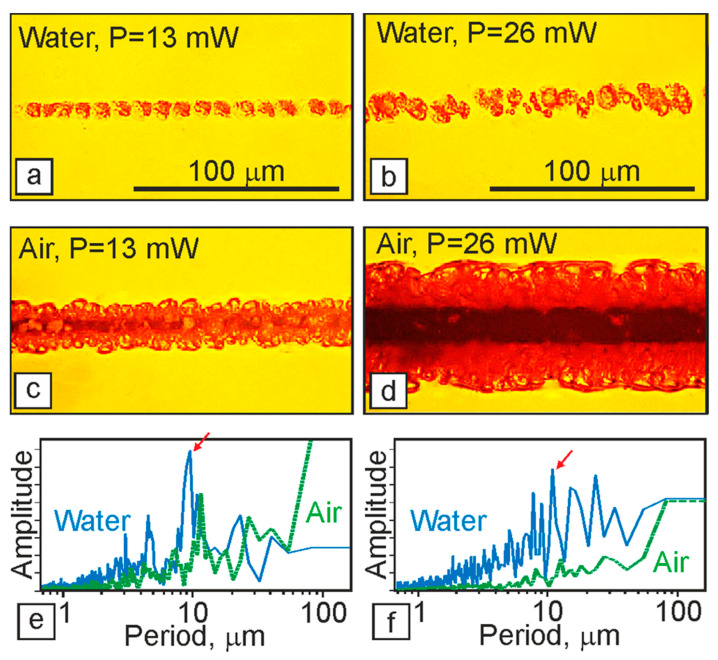
Above: (**a**,**b**) Optical photographs of fragments of track structures obtained on the surface of an OPBI film located in an aqueous and (**c**,**d**) air medium at a laser power of 13 mW (**a**,**c**) and 26 mW (**b**,**d**). Below: The spatial spectra obtained along with these structures for powers of 13 mW (**e**) and 26 mW (**f**), respectively.

In Figure 2, optical micrographs of fragments of track structures obtained on the surface of an OPBI film in air or water are presented. It can be seen that with the same parameters of laser action in the air, wider stripes are formed compared to the aqueous medium. At the same time, as can be clearly seen, these structures are continuous in the air and “dashed”, i.e., intermittent, in water. When laser “drawing” in an aqueous medium and at relatively low laser power (P = 13 mW), a linear chain of bubble formations separated by almost equal spatial intervals is observed. At higher laser powers (P = 26 mW), the structures change—the track width more than doubles due to an increase in the number of bubbles in the transverse direction.

It should be noted that during laser “drawing” in air, the formation of a dark dip in the axial region of the track, which is associated with the destruction and burnout of bubble structures in the zone of maximum intensity of the laser action, is observed, as already noted by our group [9]. With an increase in the laser radiation power, the width of this dip increases.

We believe that the effects described above (the width of the bands formed and the features of their structure) are primarily associated with differences in the heating of the polymer film surface due to changing heat removal during laser painting in different media using the same laser parameters—the thermal conductivity coefficient for air is 20 mW/(m·K), and for water, it is 600 mW/(m·K).

In Figure 2 below (Figure 2e,f) are the spatial spectra representing the spatial distribution of the intensities of the pixels along the axis of the formed microstructures in the obtained optical images. Such spectra make it possible to determine the presence of a periodic structure in the analyzed image and to identify and compare the amplitudes of the most characteristic periods. Comparing the given spectra with each other, we can note a certain similarity between the spectra for water (radiation power 13 mW and 26 mW) and the spectrum for air at 13 mW power. Therefore, several characteristic peaks emerge on all these curves, with the most intense one located in the region of 10 μm. The obtained results suggest that the mechanism of the formation of microstructures in these three cases is similar and does not strongly depend on the power density of laser radiation or the type of medium in which the polymer film is located. At the same time, the curve describing the spectrum for air at a laser power of 26 mW differs significantly from the other three described above in the absence of pronounced peaks. This effect can be associated with the fact that, in addition to the formation of bubble structures in the central part of the OPBI film, in this case, there is intense destruction of the material, accompanied by the burnout of the formed structures.

**Figure 3:**
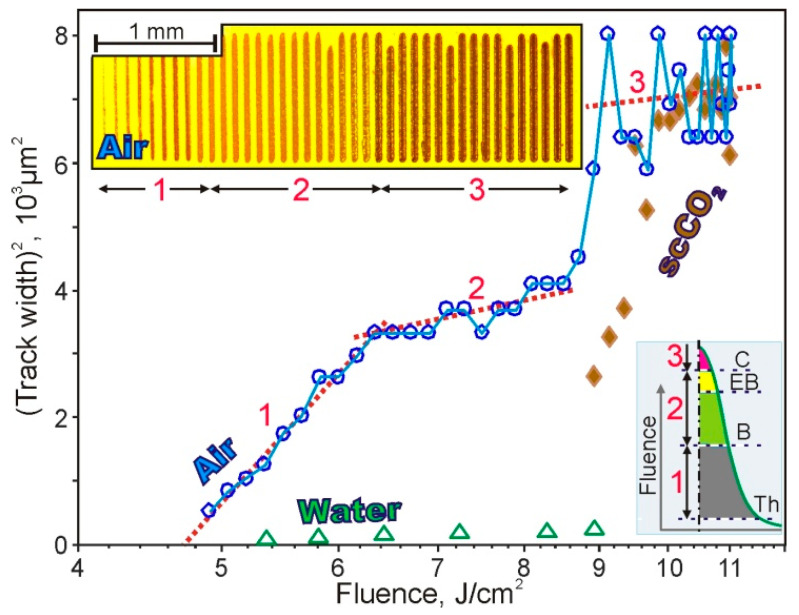
Graph of dependence of the squared width of linear tracks on the laser fluence for different media. Linear trends are plotted with red dotted lines in characteristic areas 1, 2, and 3. Insets: (top left) optical image of linear tracks at various fluences and (bottom right) qualitative representation of the fluence distribution transverse to the track with thresholds: Th—threshold, B—boiling, EB—explosive boiling of formic acid, C—carbonization of polymer.

Figure 3 shows the dependence of the squared width of the linear tracks on the laser fluence for different media. This dependence is used for Gaussian beams to determine the threshold value of the fluence and the characteristic regions, including those caused by phase transitions (see, for example, [27]).

Detailed results for air are presented in Figure 3. It can be seen that with the shape of the fluence, the width of the tracks on average increases. However, several characteristic sections are clearly distinguished on the curve, indicated in the Figures by numbers 1, 2, and 3. Section 1 is characterized by a linear dependence. The point of intersection of trend 1 with the fluence axis corresponds to a threshold value of ~2.3 J/cm^2^. The slope angles of the linear trends in Section 2 and Section 3 are much lower. The presence of phase transitions can explain this change. We believe that they are associated with the boiling and explosive boiling of formic acid in Section 2 and polymer’s carbonization in Section 3. The sharp increase in the width of the linear tracks between Section 2 and Section 3 can be explained by the fact that there is an almost complete evaporation of the near-surface formic acid by the end of Section 2.

The weight loss dependences obtained earlier using TGA show that the total loss of the solvent occurs in the temperature range 200–230 °C [12]. Some of the characteristic changes noted above can be seen in the optical photograph of the sample (inset in Figure 3). In particular, Section 3 here is characterized by the blackening of the axial region of the tracks associated with the carbonization of the polymer.

The points corresponding to the aquatic environment in Figure 3 are located near the fluence axis, which is explained by the high thermal conductivity of water. The points corresponding to the scCO_2_ environment at high fluence are in the area of the points for air. However, with decreasing fluence, the track width decreases significantly faster than in the case of air. With fluence < 9 J/cm^2^ the stripes on the OPBI surface disappear, which, in our opinion, is associated with the specificity of scCO_2_.

### 3.2. Features of the Formation of Microstructures in OPBI Samples in scCO_2_ Medium with Dissolved Precursor Molecules

The purpose of the experiments was to study the features of the laser-induced formation process of foam-like structures in the near-surface layer of polymer samples in a supercritical fluid medium, which, in its physicochemical properties, is very different from ordinary liquids. The scCO_2_ medium has approximately three-times higher thermal conductivity compared to air (>60 mW/m·K for scCO_2_) [28] with the parameters used in our experiments. In addition, it is important to note that, due to its unusually low viscosity compared with water, as well as its high diffusion properties [29], scCO_2_, together with the precursor molecules dissolved in it, quite easily penetrates into porous matrices to micron-scale depths [20].

Note that the experiments carried out in this work were also aimed at testing the possibility of realizing a one-stage process of laser synthesis of structures with new functional properties—for example, for creating plasmonic microstructures containing silver nanoparticles. Earlier, the possibility of the laser-induced synthesis of such microstructures in the near-surface layers of polymer samples of oligourethane methacrylate (OUM) was realized by a two-stage process [7,8]. At the first stage, the sample was impregnated with silver-containing Ag(hfac)COD precursor molecules dissolved in scCO_2_. Then, at the second stage, the samples were irradiated in the air with continuous UV or visible laser radiation. This led to the formation of microstructures in the irradiated regions using the known mechanism of photolysis of precursor molecules and the subsequent self-assembly of Ag atoms into nanoparticles (AgNPs) [8]. As applied to the OPBI film samples used in this work, the formation of plasmonic microstructures was previously a multistage process. In particular, in this rather dense (compared to OUM) polymer matrix, to obtain regions with increased porosity in the near-surface layer, at the first stage, extended foam-like structures were created by the method of laser “drawing” [9]. Then, the samples were treated in scCO_2_ with dissolved molecules of the silver precursor. For the more efficient reduction in silver atoms from the precursor with the subsequent formation of Ag NPs, polymer samples were kept at a temperature of 140 °C [8] or were exposed to UV. In this situation, both of these processes can be attributed to the final stage. Note that the elements of this technique (production of foam-like track structures and supercritical impregnation of a polymer matrix with a precursor) were used to create extended luminescent structures upon impregnation of OPBI films with europium-containing Eu (dbm)·3H_2_O molecules [16].

**Figure 4:**
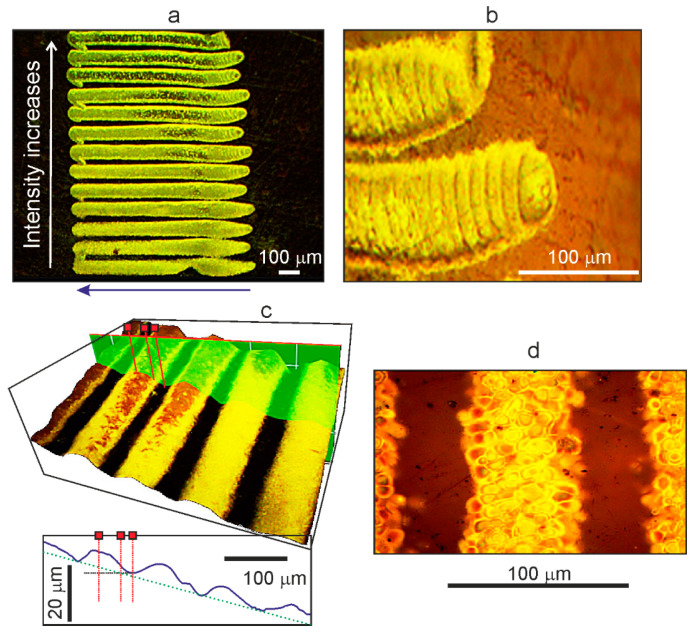
Images of linear microstructures obtained by laser “drawing” on the surfaces of OPBI films in scCO_2_ medium with dissolved Ag(hfac)COD molecules. (**a**) Photograph from an optical microscope of formed tracks (in reflected light). (**b**) Photograph of the initial sections of two of these tracks (in transmitted light). (**c**) 3D image for several tracks (in reflected light) shown in Figure 4a. (**d**) For comparison, a photograph of a part of a track obtained on a film in an environment of pure scCO_2_ (in transmitted light) is presented.

Figure 4 shows optical photographs of linear microstructures obtained by laser “drawing” on the surfaces of OPBI films in a high-pressure cell with scCO_2_, with dissolved molecules of the Ag(hfac)COD precursor. Microstructures in the form of tracks were formed at a constant laser power P = 30 mW, but at different radiation intensities in the laser spot. Figure 4a demonstrates photographs of 13 tracks obtained with a gradual change in intensity. The first tracks from above, “drawn” with more rigid focusing of the laser radiation on the film surface, have a clearly pronounced, darker paraxial region, which gradually disappears as the beam is defocused.

The curvatures of the arcuate shape of the initial and final sections in the tracks observed in the figure are associated with a slight inconsistency in the movement of the motorized slides and the laser shutter. In addition, the arcing that appears may be a result of the deflection of the beam under the influence of refractive index gradients due to the arising local convection flows for heated fluid layers [28] when they transfer thermal energy in the zone of laser radiation absorption by a mass of polymer material.

Further analysis of microstructures revealed some features in the mechanism of their formation.

The first feature is associated with a rather large width of the formed tracks—80–90 µm, which is in good agreement with the width of similar tracks formed in pure films in air. Since scCO_2_ medium has higher thermal conductivity compared to air, it was expected that the structures width in the case of a supercritical medium would be smaller. We believe that the more efficient formation of microstructures in scCO_2_ with dissolved precursor molecules occurs due to the synergistic effect of several factors. First, the increased temperature in the scCO_2_ environment, as compared to experiments in water and in air, can lead to more efficient bubble formation due to additional thermal stimulation from the release of formic acid molecules from weakly bound states in the polymer matrix [10,12]. Second, changes in the physical properties of the polymer in the scCO_2_ medium, namely the resulting plasticization of the matrix (stretching and loosening of polymer chains) [30], can accelerate the self-assembly of Ag NPs during the photolysis of precursor molecules. In this case, the formed nanoparticles with plasmon absorption in the wavelength region of laser action (in the region of 400–500 nm [31]) themselves contribute to additional heating of the polymer matrix, which contributes to the more efficient decomposition of the precursor.

The second feature is that at the beginning of each track formed in an environment of scCO_2_, there is also a rather long section (up to 10% in length) where the process of “drawing” the track gradually reaches a stationary mode (Figure 4b). This fact distinguishes them from structures formed in the air with comparable irradiation parameters. In the initial part of the track, transverse periodic annular formations are clearly visible, separated by dark gaps. The width of the rings in the direction of the beam movement gradually decreases from ~35 μm to ~10 μm or less. We believe that their appearance is associated with the same thermophysical processes that were noted above for the case of the formation of periodic bubble clusters in “drawn” tracks on films in water (see Figure 2).

As shown in Figure 4a, when approaching a more rigid focusing in the paraxial region of the track structures, an increase in the area of the darkened regions is observed (7 tracks in the upper part). The appearance of such areas can be explained, as in the case of structures formed in the air (Figure 2d for a power density of ~3 kW/cm^2^), by the destruction and burnout of bubble structures in the zone of maximum laser intensity.

**Figure 5:**
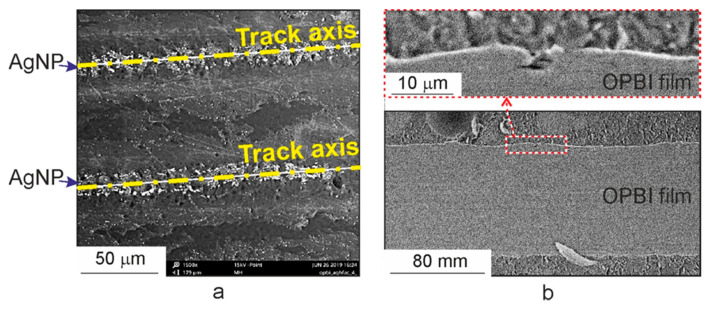
SEM images of tracks on the surface of the OPBI film sample: (**a**) A section of the film surface with formed tracks; (**b**) A cross-section of the OPBI film with a formed track. Laser “drawing” was carried out in a scCO_2_ environment with a dissolved silver precursor.

Further studies of tracks with pronounced darkening along the axis were carried out using SEM. In Figure 5, it can be seen that the central axial region of the track with clusters of light dots corresponds to destroyed bubble formations observed in optical images in the form of a darkened region. Similar images in electronic photographs for “drawn” structures in clean films in the air were presented in [9], where the dark central part of the track with destroyed bubble formations is clearly visible. In contrast to those structures, in our case (Figure 5a), along the entire length of the scanned region, clusters of Ag NPs, presented as light dots, are observed. The presence of Ag NPs with a silver content of 0.5–1.0 wt% in these track regions is confirmed by the results of an elemental analysis performed using EDX. Figure 5b shows SEM photographs for thin cross-sections of irradiated samples of OPBI films in the region of track formation under conditions of tight focusing of laser radiation. The photograph shows the formation of cavities up to 4 µm deep in the near-surface layer of the polymer film at the site of radiation exposure.

In general, the results presented in this section are the first step towards the study of the processes of laser formation of microstructures in polymer matrices in the SCF medium.

### 3.3. Contact Formation of Microstructures Using OPBI Polymer Film Samples

In this work, we also carried out experiments to assess the possibility of laser “drawing” of microstructures through a backside surface of a quartz plate lying on the surface of a polymer film sample. Experiments on the laser “drawing” of microstructures were carried out in the air. They were actually carried out under contact conditions between the film and a medium with a high heat capacity. We used samples of OPBI films preliminarily impregnated with known molecules of a silver precursor in a scCO_2_ medium to solve this problem. For some comparative experiments, four-component OUM-based films, which were similarly preliminarily impregnated, were used [30]. For the experiment, the obtained samples were tightly fixed between plates of quartz glass with a size of 20 × 20 mm and a thickness of 1 mm, after which they were irradiated with laser radiation.

**Figure 6:**
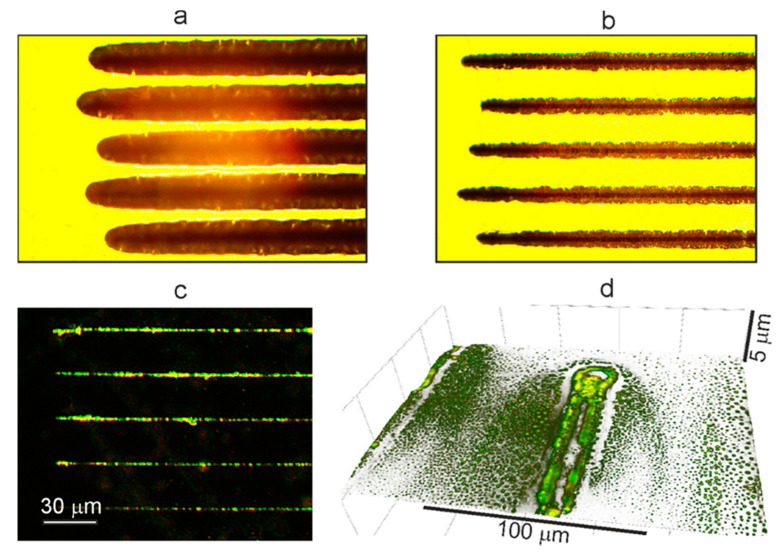
Photographs from an optical 3D microscope of fragments of laser “drawn” track structures on the surface of the OPBI film and their “prints” on the surface of a glass plate. (**a**) Tracks on the film, obtained in the air without contact with a glass plate, observed in transmitted light; (**b**) Tracks on the film, obtained by contact with the glass plate, observed in transmitted light; (**c**) “Prints” of tracks on a glass plate after laser drowning on film; (**d**) 3D image of the fragment of the “prints” on the glass from the track, “drawn” on the OPBI film. Contrast images of “prints” are observed in dark field mode by scattered light.

The experiments have shown that when the glass is in close contact with the film surface, for the formation of structures, laser powers are required that are 25–30% higher than in the case of irradiation of a film freely located in the air. It should be noted that at such powers, the width of the film tracks without quartz plates can be 2–3 times larger than in the case of the formation of structures through a quartz plate (compare Figure 6a,b). This fact, as in the above results of the formation of structures in films in water or air (Figure 2), is explained by the difference in heat removal: the thermal conductivity coefficient for air is 20 mW/(m·K), and for quartz, it is 1 W/(m·K). In addition, it should be considered that the very presence of a quartz plate above the film also leads to a decrease (by at least 8%) in the value of the incident energy flux due to reflection from the two surfaces.

Figure 6 shows optical photographs and topographic reliefs obtained on a 3D microscope for OPBI film samples with “drawn” fragments of structures and their “prints”, i.e., traces of these structures left on the surface of the pressed plate. The topographic 3D image of the initial fragment of the “prints” (Figure 6d) demonstrates the complex nature of the deposition of the molten polymer material, which escapes in the form of microdroplets from the forming track during laser irradiation. It can be seen that, in addition to the predominant deposition of the material directly above the surface of the track structure, a large number of microdroplets settled at a sufficiently large distance from the centerline of the track, practically covering the glass surface at a distance of hundreds of microns (Figure 6d).

This effect can occur if, during exposure to laser radiation, the so-called explosive boiling process develops in the near-surface layer of the OPBI polymer film. This process leads to the appearance of microstructures on the surface of a tightly pressed quartz plate. The specifics of the explosive boiling mechanism with the participation of formic acid dissolved in the polymer matrix will be considered in the last section of this article. As a result of the development of such processes, diffuse accumulations of microdroplets appear on the surface of a quartz plate. Where, at the beginning of the track formation, these processes have not yet developed due to the short heating time, the formation of microdroplet structures at a distance from the main “print” of the track is practically not manifested, and the entire mass of the evaporated polymer is concentrated inside the track contour (Figure 6d). In the formation of such an extensive microdroplet distribution on the contacting surface of the glass plate the elastic deformation characteristics of the OPBI films themselves apparently play an important role.

**Figure 7:**
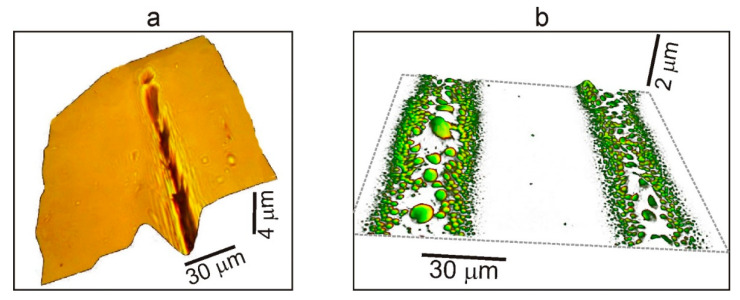
(**a**) Photographs from an optical 3D microscope of fragments of laser “drawn” track structures on the surface of the OUM-based polymer sample. The formation of an elongated depression in the direction of the laser beam is illustrated; (**b**) 3D image of the fragment of the “prints” on the glass from the track, “drawn” on this sample.

Indeed, if, instead of the OPBI film, we take a more plastic OUM-based polymer material [32] that does not contain dissolved formic acid molecules, then the picture of the process will be somewhat different (Figure 7b). In this case, a bright contour is also clearly observed in the photograph of the initial fragment near the “print” of the track on the glass, but no microdroplet distribution outside it is observed. In this situation, the OUM-based matrix can be considered a single phase concerning the OPBI matrix. The dissolved formic acid represents a different phase in the OPBI matrix that boils up during irradiation. As a result, there are no effects associated with explosive boiling processes and stimulating the development of microdroplet spraying of the evaporated material. In this case, the appearance of an elongated depression in the course of the movement of the laser beam without any traces of the formation of bubble structures is observed in the OUM-based polymer sample (Figure 7a).

In regard to the appearance of the bright contour composed of polymer material observed in both cases (Figure 6d and Figure 7b), which actually corresponds to the track boundaries, this is most likely associated with the manifestation of the Marangoni effect for polymer matrices heated to a liquid state under the action of laser radiation [33]. In this case, the evaporated polymer masses brought to the glass surface are deposited on the surface of the quartz plate. Furthermore, the cooling masses move from the hot central region to the colder region at the periphery; there, they freeze, forming a track outline. We have already considered similar effects in the formation of ring structures under the action of continuous laser radiation in ethyl methacrylate copolymer films with silver nanoparticles [34].

Thus, the experimental results presented in this section demonstrate some features of the mechanism of removal and condensation of evaporated polymer material in a similar method of laser action on polymer films, and show some new possibilities for obtaining functionalized polymer microstructures on substrates of another material. It should be noted that the approach we propose is a variation of the well-known laser-induced backward transfer (LIBT) technology for printing polymer structures on substrates using pulsed laser radiation (see, for example, [24]). This approach will have advantages compared to LIBT technology due to the use of simpler and cheaper continuous UV laser sources. It seems to us that this advantage will manifest itself only when using polymers with a high level of absorption at the wavelength of laser radiation.

### 3.4. Raman Spectra in OPBI Films with Surface Microstructures

The previous sections show that when the OPBI polymer films are exposed to laser radiation, it is possible not only to form foam-like structures on their surfaces but to create “prints” of these structures on the transparent materials that are in contact. Similar processes occur when the polymer surface is heated by several hundred degrees [12]. As the experiment shows, at temperatures > 500 °C in the zone of laser action on the surface of the OPBI film, destruction of the polymer and its carbonization can occur. These effects can be seen in the Raman spectra obtained by probing individual points with an area of several square microns in the darkened paraxial regions of the “drawn” track structures.

**Figure 8:**
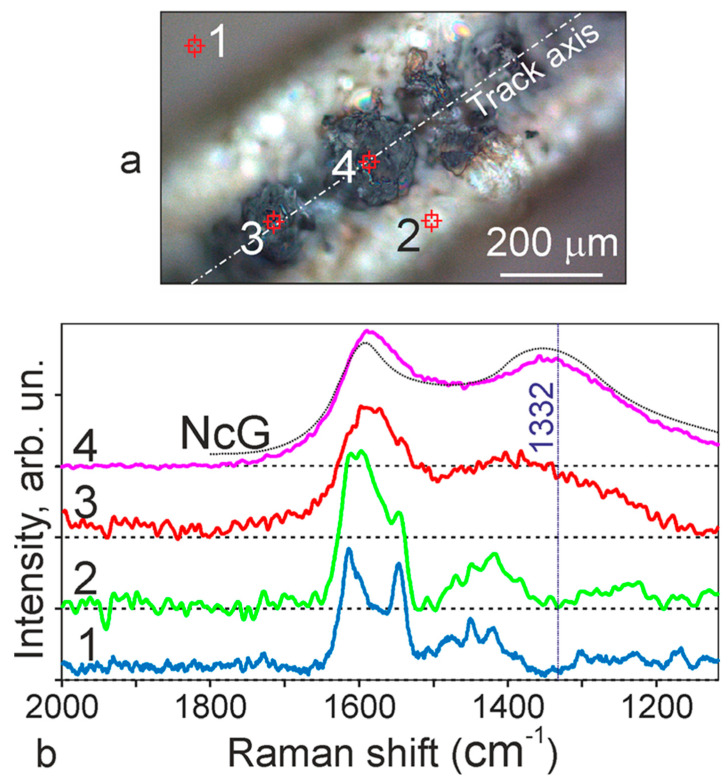
(**a**) Photograph of the OPBI tape with a “drawn” section of the track and marked points; (**b**) Raman spectra corresponding to these points: spectrum 1—outside the track, spectrum 2—in its peripheral region surrounding the bubble formation zone, spectrum 3—in the central paraxial region with destroyed bubbles, spectrum 4—in the central paraxial region, another point corresponding to a microparticle of carbon. The figure also shows the spectrum in a dotted curve for a control sample of graphite nanoparticles [35]. The dotted vertical line shows the position of the diamond feature line.

Raman spectra were measured on samples of OPBI polymer film irradiated in air. We analyzed the track (Figure 8a), which has the most pronounced dark area along its axis, corresponding to the zone of maximum destruction of bubble formations [9]. Figure 8b shows spectra from several microregions of the OBPI film with tracks. Spectrum 1 refers to the region not exposed to laser action. Spectrum 2 refers to the peripheral regions of the track, i.e., from the surfaces of formed and not destroyed bubbles. Spectra 3 and 4 were obtained for points in the central paraxial region of the track with destroyed bubbles. For comparison, the dashed line in this figure shows the reference spectrum for graphite nanoparticles [35].

Note that when measuring the Raman spectra in individual regions of the film, an intense luminescence emission of the polymer matrix from the exciting radiation was recorded, especially in the regions previously exposed to laser action during the formation of structures. The nature of this emission in films based on benzimidazole was discussed in several works [9,10,17,18]. According to our research [9,10], the luminescence mechanism is associated with singlet-triplet π ⇾π * transitions in OPBI molecules. The presence of a powerful luminescent signal makes it difficult to record Raman spectra from these samples. For a more convincing interpretation and qualitative comparison, all spectra presented in Figure 8b are shown with the background luminescence signal subtracted.

It can be noted that there is a certain similarity between the spectra presented, but significant differences are also noticeable. Spectrum 1, corresponding to an unirradiated region of the OPBI film, consists of a series of rather narrow bands, among which two regions are clearly distinguished in the form of a complex peak at 1613 cm^–1^ and a peak with a maximum at 1546 cm^–1^. This group of peaks in their position can be identified according to [24] as bands related to benzimidazole stretching vibrations of structures C=C/C=N. The second group of bands is outlined in the region of 1473–1419 cm^−1^. It is five-times lower in intensity than the first and belongs to the same type of structure, C=C/C=N.

Spectrum 2 was recorded in an already formed track at its periphery in the region of bubble formation. It differs from the previous one by the noticeable transformation of the components within the 1543–1613 cm^−1^ continuum, associated with the redistribution of the intensities of its individual components. We believe that such redistribution is due to the development of strained (stretched) bonds in the molecular chains that make up the OPBI matrix while forming the surface of individual bubbles.

The case corresponding to the paraxial region marked in Figure 8a shows spectra 3 and 4. Moreover, for spectrum 4, in the region under study, background luminescence was not initially observed, which made it possible to obtain a clear spectrum with all its components in the form of two intense and rather wide (50–60 cm^−1^) peaks, with maxima at 1346 and 1582 cm^−1^. Both peaks, judging by their parameters, are very close to the characteristics of a mixture of amorphous carbon and graphite, which appear in organic materials after their high-temperature treatment at 900 °C and above [35,36,37,38]. Note that a situation similar to our experiments was considered in [24]. In this case, clusters of amorphous carbon or graphite appeared in polyimide films under the action of focused UV radiation, which has practically the same lines in the Raman spectra. As stated in this work, at sufficiently high concentrations of these clusters on the plane, it is possible to form structures with high electrical conductivity from them.

### 3.5. Model of Laser-Induced Formation of Microstructures in OPBI Films

**Figure 9:**
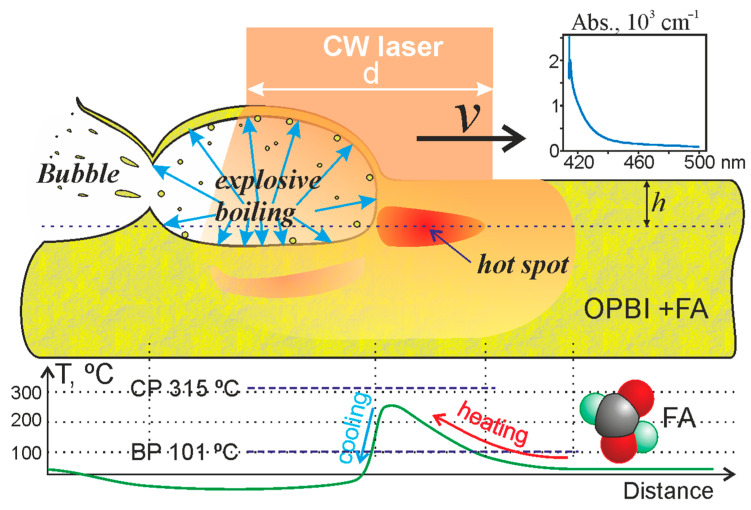
A qualitative model of the formation of periodic bubble structures in the near-surface layer of OPBI when the laser beam moves over the sample’s surface. Top right: absorption spectrum of OPBI. Bottom: a model graph of the temperature distribution in the near-surface layer of the polymer when the laser beam moves. The horizontal dashed lines in the graph represent the boiling point BP and critical temperature CP for formic acid.

Figure 9 presents a qualitative model illustrating the formation process for periodic bubble foam-like structures in the near-surface layers of samples of polymer films from OPBI during laser “drawing”.

We believe that laser radiation with a wavelength λ = 405 nm, focused on the sample surface into a spot of diameter d, moves along its surface at speed V. With a sufficiently large absorption coefficient at this wavelength (k_abs_ > 2000 cm^−1^, as can be seen from the graph of the absorption spectrum for OBPI in Figure 9), laser action will lead to heating of the polymer mass. In this case, the main part of the laser radiation energy will be absorbed in a thin, near-surface layer with a thickness of 1/k_abs_ < 5 μm, which will lead to its gradual heating to high temperatures. Since this near-surface layer from above borders on the heat-conducting medium, the maximum temperature in the layer will be observed at a certain depth h (“hotspot” area, Figure 9). In addition, due to light scattering in the near-surface layers of the polymer, even in the absence of contact with the heat-conducting medium, the maximum absorption will occur at some distance from the surface [39].

Upon laser heating, a group of formic acid (FA) molecules will begin to detach from the weakly bound molecules of polymer chains [12] and, while diffusing, condense (accumulate) in the regions of structural microdefects of the polymer matrix and microbubbles [9,12,40] As a result of a further increase in temperature, upon reaching a threshold close to the critical temperature of formic acid (T_c_ = 315 °C), explosive boiling will occur [41]. In this case, the liquid in the microvolumes will quickly transform into a gas compressed to high pressure (close to the critical pressure Pc = 5.81 MPa [42]) with liquid microdroplets.

Since the volume of this gas, compressed in numerous microcavities, surrounds the polymer material, which is softened due to the increased temperature, the microbubbles will begin to expand sharply (the so-called thermocavitation effect will occur [43]). With this expansion, they will break and grind the polymer material and eventually form one or more expanding macrobubbles. Such flying microscopic particles of polymer material are conventionally shown in Figure 9. Note that, due to the proximity to the polymer surface, the expansion of macrobubbles will not occur uniformly in all directions but mainly upward. As a result, a bulging area forms on the material’s surface: one or more polymer bubbles, which are clearly visually observed on polymer films (see, for example, Figure 2). For the parameters used, the height of such bulges can be several hundred nanometers (see, for example, Figure 6b).

It should be noted that during the growth of the bubble, the pressure and temperature inside it will rapidly drop. Their values for the ideal gas are well described by the dependencies: $PV^{\gamma }=const$ and $TV^{\gamma -1}=const$, where *P* is pressure, *V* is the volume of the bubble, *T* is temperature and γ is the adiabatic index (for a monatomic ideal gas γ = 5/3, and for a diatomic gas γ = 7/5). Therefore, when the volume of the bubble increases by 100 times, the pressure in it will be ~ 5 × 10^−4^ from the initial one, and the temperature will be 0.05 from the initial one. If we use critical parameters as initial parameters, then, with such an expansion, the pressure will drop to ~0.03 atm, and the temperature to 30 K. In the real case, the cooling of the internal volume due to heat transfer from the walls of the bubble will not be so significant. The above-described cooling effect, which occurs during the formation of bubble structures, was previously confirmed experimentally in [12], in which the temperature on the surface of an OBPI polymer film was measured using a thermal imager with a micro-lens during laser action without scanning. Experimental data have shown that as the bubble grows, the temperature on its surface drops by several tens of degrees. Figure 8 shows a model graph of the temperature distribution in the near-surface layer of the polymer after the formation of the next bubble, which demonstrates a significant decrease in temperature in the inner volume of the bubble.

On the other hand, the heat flux described above, directed inside the expanding bubble, will also lead to the cooling of the polymer material adjacent to the bubble surface (arrow “cooling” in Figure 9). Therefore, the next act of explosive boiling will not occur immediately after the previous one, but after some time Δ*t*. During this time, the laser spot will move to a distance *v·*Δ*t*, where *v* is the scanning speed, so that the next bubble will appear at this distance from the previous one. This means that, due to the described processes, a chain of bubbles will be formed (see Figure 2).

The features of bubble microstructures formed on the polymer surface, in addition to the parameters of laser action (power density of laser radiation, speed of movement of the laser beam) and the optical and thermophysical characteristics of the material, will obviously strongly depend on the environment. With an increase in heat transfer to the surrounding space, heating will occur more slowly. In this case, explosive boiling will occur when a lower superheat is reached; therefore, the pressure surges will be less. Taking into account that the polymer surface, in this case, will be heated to lower temperatures, its plasticity will be lower. Moreover, the combination of less plasticity and less internal pressure will result in smaller bubbles. In this case, they will appear at a greater distance from each other. This is precisely the picture observed when the air medium is replaced by water (Figure 2): the bubbles significantly decrease in size, and the distance between them increases. A similar picture is observed when a polymer film is applied to a glass plate (compare Figure 6a,c), as a result of which, the width of the structures formed on the OPBI surface significantly decreases.

Also note that in similar cases, under pulsed laser action on polymers, microbubbles can form not only due to heating and boiling of the liquid contained in the polymer and its phase transition but also at the action of negative pressure arising in the process of pulsed laser heating of the material [14,15]. In this case, the formation of microbubbles and microfoam can occur at a temperature below the boiling point of the liquid due to a sharp decrease in pressure during the passage of a compression wave associated with short-pulse laser heating. It cannot be ruled out that in our case, microbubbles are forming in the polymer matrix by this mechanism [14] during the explosive boiling of formic acid, which is always accompanied by the generation of powerful pressure surges [43].

Note that the bubble shells may lose their integrity during expansion, and the bubbles will burst at high fluences. In this case, instead of bulges on the polymer surface, a channel with depressions and uneven, raised edges is formed in the axial region of the track. If the film at this time is covered with a transparent plate, then the material microparticles scattered during these processes will settle on its surface and form a kind of “imprint” of the structure formed on the polymer film (Figure 5e and Figure 6d). Increased fluences can also lead to the destruction and burnout of bubble structures (see upper tracks in Figure 4), primarily occurring in the paraxial zone with the maximum laser radiation intensity [9].

It is also worth noting that the initial destruction of bubble polymer structures under the action of continuous laser radiation occurs in the process of explosive boiling, which develops in the near-surface layers of the OPBI polymer matrix. In this case, we believe that a rupture of relatively weak bonds between individual polymer chains occurs [44].

As for the direct breaking of bonds upon absorption of light quanta, in our case, multiphoton processes are absent due to the low intensity. The energy of a quantum (about 3 eV) is enough only to break the weakest bonds in the polymer chain (of the π_c=c_ bond type [45]). Thus, it can be expected that when strained bonds appear due to the appearance of microbubbles, taking into account additional heating and direct laser action, a rupture of bonds between the benzene and imidazole rings of neighboring units may occur (see Figure 1 in [10]).

Changes in the position and shape of the Raman bands in the region of 1613 cm^−1^ associated with benzimidazole stretching vibrations of structures C=C/C=N during the transition from the initial matrix to the stressed structures in the bubbles (Figure 8) can serve as an indirect confirmation of the development of such a process.

## 4. Conclusions

The paper presents results regarding the laser formation of microstructures on the surfaces of polymer film samples of OPBI, a rigid polymer matrix with formic acid dissolved in it. Laser “drawing” of microstructures was carried out using focused continuous laser radiation with λ = 405 nm in three media: air, water and a scCO_2_ solution. At the same time, experiments in the scCO_2_ environment were carried out with the molecules of the silver precursor Ag(hfac)COD dissolved in it, which made it possible to form microstructures containing plasmonic silver nanoparticles.

It was shown that in all media, the formed microstructures consisted of microbubbles, and the pronounced spatial periodicity of their formation was observed. The geometrical dimensions of the microstructures and periodicity largely depended on the environment in which they were formed, which was explained by the change in heat-conducting properties when the medium was changed. A model was proposed to explain the formation of such structures and the appearance of the observed periodicities in a two-phase material. The key point of this model is the participation of the photoinduced mechanism of explosive boiling of formic acid dissolved in the polymer matrix.

Using Raman spectroscopy, it was shown that in the region of the laser action, the polymer was modified, leading to high fluence to form amorphous carbon and graphite.

The possibility of creating microstructures in the form of prints on the back surface of a quartz plate that was in close contact with the polymer sample during the laser formation of microstructures on the sample surface was demonstrated. A mechanism for the formation of such structures was proposed.

Thus, the work demonstrates new approaches that make it possible to create plasmonic, luminescent and, potentially, electrically conductive microstructures on the surfaces of OPBI polymer films using continuous laser radiation with λ = 405 nm.

The proposed methods of the laser “drawing” of structures in such polymer films, and the method of obtaining prints of these structures on other transparent materials with their further improvement, can be used in various branches of polymer microelectronics.

## Data Availability

The data presented in this study are available on request from the corresponding author.
